# Bioactive Polysaccharides from Blue Honeysuckle Berries: Structural Properties and Digestive Enzyme Inhibition Activities

**DOI:** 10.3390/ijms27072982

**Published:** 2026-03-25

**Authors:** Na Ding, Juan Sun, Jin Li, Junwei Huo, Yan Zhang

**Affiliations:** 1 College of Horticulture and Landscape Architecture, Northeast Agricultural University, Harbin 150030, China; 2 National-Local Joint Engineering Research Center for Development and Utilization of Small Fruits in Cold Regions, Northeast Agricultural University, Harbin 150030, China; 3 Heilongjiang Green Food Science Research Institute, Northeast Agricultural University, Harbin 150030, China

**Keywords:** haskap, polysaccharide, monosaccharide composition, pancreatic lipase inhibition

## Abstract

The physicochemical and bioactive properties of polysaccharides from blue honeysuckle (*Lonicera caerulea* L.) berries were comprehensively investigated. Fourier transform infrared spectroscopy confirmed that both samples were acid heteropolysaccharides. High-performance liquid chromatography detailed a monosaccharide profile of arabinose, galacturonic acid, rhamnose, galactose, and glucose in specific molar ratios. High-performance gel permeation chromatography further revealed variations in molecular weight and distribution among the samples. Functionally, the polysaccharides exhibited significant in vitro antioxidant capacity. For the first time, the polysaccharides are shown to inhibit pancreatic lipase, in addition to α-amylase and α-glucosidase, demonstrating potent inhibitory activity with low IC_50_ values (2.80 ± 0.12 mg/mL). This bioactivity, particularly toward lipase, was correlated with structural properties such as monosaccharide profile and molecular weight. These results highlight the potential of these polysaccharides as functional food ingredients for managing hyperglycemia and obesity and provide novel insights into their structure–activity relationships.

## 1. Introduction

In the family Caprifoliaceae, blue honeysuckle (*Lonicera caerulea* L.) is a deciduous shrub widely distributed across northern China, Japan, Russia, as well as Northern and Eastern Europe, including Finland, Sweden, and Poland [1,2]. Recognized as a fruit with ‘homology of medicine and food’, it is valued for its rich content of bioactive compounds that contribute beneficially to human health [3]. Among these, polysaccharides represent a major class of functional macromolecules. Polysaccharides constitute a significant category of key bioactive secondary metabolites in blue honeysuckle berries, and several studies have examined their functional characteristics in recent years. Research has focused on purified, degraded, fermented, and selenized polysaccharides. Pei et al. [4] reported that purified polysaccharides of blue honeysuckle pomace exhibited high thermal stability along with antioxidant, hypoglycemic, and hypolipidemic activities. Ma et al. [5] showed that two degraded polysaccharides displayed higher reducing sugar content and improved antiglycation and starch-digesting enzyme inhibition. Pei et al. [6] further demonstrated that probiotic fermentation reduced molecular weight, elevated galacturonic acid content, and enhanced free radical scavenging and α-amylase inhibition activities. Shao et al. [7] found that selenized polysaccharides had stronger antioxidant capacity, lower molecular weight, and enhanced acetylcholinesterase (AChE) inhibition activity. Additionally, Fu et al. [8] identified an acidic heteropolysaccharide with high uronic acid content and potent inhibition activities against α-amylase and α-glucosidase. These findings underscore the fundamental principle that the structure of polysaccharides dictates their biological function. However, the existing literature lacks not only a detailed characterization of the inherent structural properties but also, in particular, a precise understanding of monosaccharide composition and its connection to specific activity mechanisms.

Although blue honeysuckle polysaccharides have been widely studied, their inhibitory activities against digestive enzymes remain insufficiently explored. Digestive enzymes include proteases, lipases, cellulases, and starch-digesting enzymes. Among these, inhibition of α-amylase, α-glucosidase, and pancreatic lipase is recognized as a promising means of controlling diabetes by delaying glucose release and combating obesity by reducing fat absorption [9]. Previous studies have confirmed that blue honeysuckle polysaccharides can inhibit starch-digesting enzymes, suggesting their potential as natural hypoglycemic agents due to their multimodal mechanisms and favorable safety profile [5]. For example, a comparative analysis of fermented and unfermented blue honeysuckle polysaccharides revealed distinct inhibition activities, with the highest inhibition activities for *Saccharomyces cerevisiae* W5-fermented polysaccharides (71.04 ± 1.89%) and for unfermented polysaccharides (97.73 ± 1.22%) [4]. Ma et al. [5] further demonstrated that oxidative degradation at 50 °C and 70 °C demonstrated a marked inhibition of α-amylase and α-glucosidase activities, reaching values of 63.85 ± 1.06% and 68.03 ± 0.85%, compared with 46.40 ± 0.77% and 42.25 ± 1.08% of the original polysaccharides. Moreover, considerable inhibition rates of 58.94 ± 3.65% against α-amylase and 67.42 ± 3.69% against α-glucosidase were investigated for the purified pomace polysaccharides from blue honeysuckle [4]. However, research in this area has been largely focused on reporting inhibition rates, with a notable gap in the elucidation of the underlying structure–activity relationships, especially regarding the contribution of specific properties like monosaccharide composition. More critically, the potential of these polysaccharides to inhibit pancreatic lipase, a key target for reducing fat absorption, has never been investigated. This represents an unexplored aspect in understanding their full bioactive potential. Therefore, a comprehensive study that not only evaluates the inhibition of these three key digestive enzymes but also systematically links the activities to detailed structural parameters, such as monosaccharide composition, is essential to advance the functional application of blue honeysuckle polysaccharides.

The study aimed to: (1) systematically characterize the structural properties of blue honeysuckle polysaccharides, particularly their monosaccharide composition; (2) comprehensively evaluate their bioactivities, including antioxidant capacity and α-amylase, α-glucosidase, and pancreatic lipase inhibition activities; and (3) elucidate the structure–activity relationships by correlating structural properties with bioactivities.

## 2. Results

### 2.1. Chemical Composition of Blue Honeysuckle Polysaccharides

**Figure 1:**
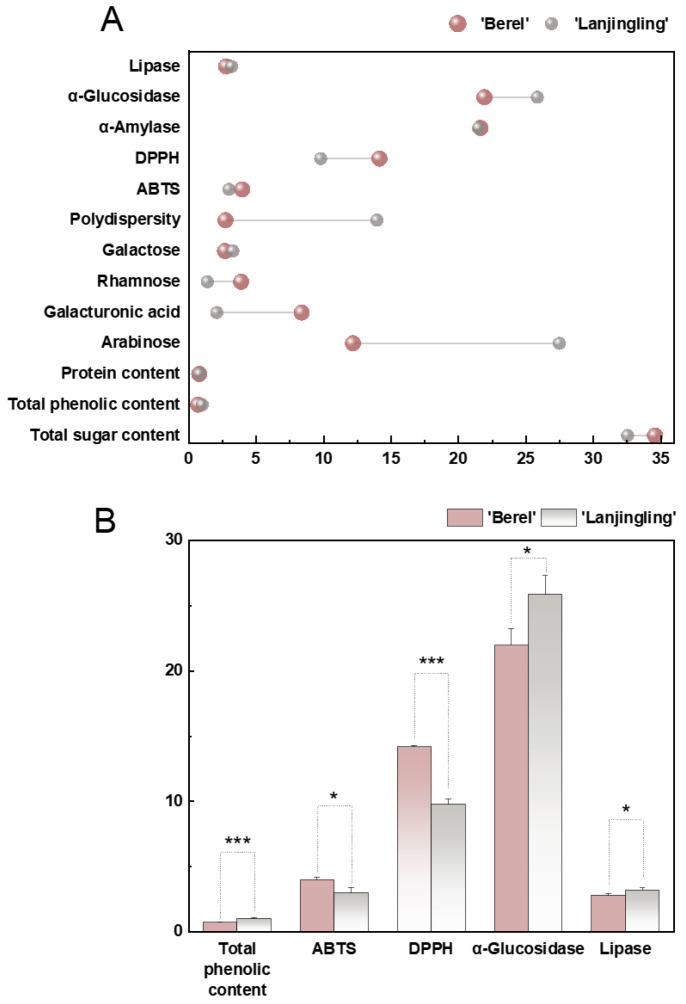
(**A**) Physicochemical properties and bioactivities of blue honeysuckle polysaccharides including total sugar content (%), total phenolic content (%), protein content (%), monosaccharide composition (molar ratio), polydispersity (Pd), ABTS^+^ (mg TE/g DW), DPPH (mg TE/g DW), α-amylase IC_50_ (mg/mL), α-glucosidase IC_50_ (mg/mL), and lipase IC_50_ (mg/mL); (**B**) five properties, with significance calculated in triplicate and shown in mean ± SD (* *p* < 0.05 and *** *p* < 0.001).

The total sugar content, total phenolic content, and protein content were quantified to characterize the chemical composition of purified blue honeysuckle polysaccharides. After dialysis using a 7 kDa membrane for two days, the polysaccharides extracted from ‘Berel’ and ‘Lanjingling’ showed comparable sugar contents of 34.59 ± 1.37% and 32.56 ± 3.28%, respectively. As shown in Figure 1A and Table 1, most free proteins were removed using the Sevag method, leaving only trace amounts in the ‘Berel’ and ‘Lanjingling’ polysaccharides (0.81 ± 0.08% and 0.82 ± 0.05%). In addition, despite using the same ethanol washing procedure to eliminate phenolic compounds, the total phenolic content in the ‘Berel’ polysaccharides (0.72 ± 0.04%) was significantly lower than that in ‘Lanjingling’ (1.03 ± 0.03%), suggesting a stronger polysaccharide–phenolic interaction in ‘Berel’.

### 2.2. FT-IR Spectrum of the Blue Honeysuckle Polysaccharides

**Figure 2:**
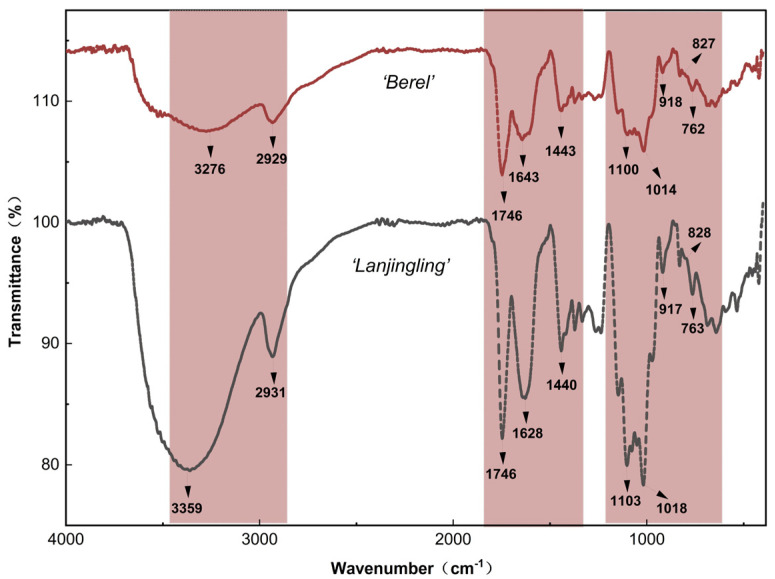
Fourier transform infrared (FT-IR) spectra of ‘Berel’ and ‘Lanjingling’ polysaccharides.

FT-IR spectroscopy was used to confirm the chemical bonds and functional groups present in the polysaccharides from the ‘Berel’ and ‘Lanjingling’ cultivars. As shown in Figure 2, both cultivars displayed largely similar characteristic absorption peaks, which could be interpreted as follows. The broad bands at 3359 cm^−1^ and 3276 cm^−1^ represented strong O-H stretching [6]. Absorption peaks at about 2931 cm^−1^ and 2929 cm^−1^ are indicative of C-H stretching vibrations [10]. Together, these O-H and C-H signals reflect typical vibrational features of glucose units. A distinct band at 1746 cm^−1^ was associated with C=O stretching, while the peaks at 1643 cm^−1^ and 1628 cm^−1^ were correlated with COO- vibrations [5].

In addition, the peaks near 1443 cm^−1^ and 1440 cm^−1^ signified uronic acid residues, consistent with previous reports [11]. The bands at 1103 cm^−1^, 1100 cm^−1^, 1018 cm^−1^, and 1014 cm^−1^ indicated that the polysaccharides exist predominantly in pyranose form [5]. Four bands at 763 cm^−1^, 762 cm^−1^, 828 cm^−1^, and 827 cm^−1^ were presented as α-terminal epimer, and the other two bands at 917 cm^−1^ and 918 cm^−1^ were shown as β-terminal epimer [12].

### 2.3. Monosaccharide Composition Analysis

**Figure 3:**
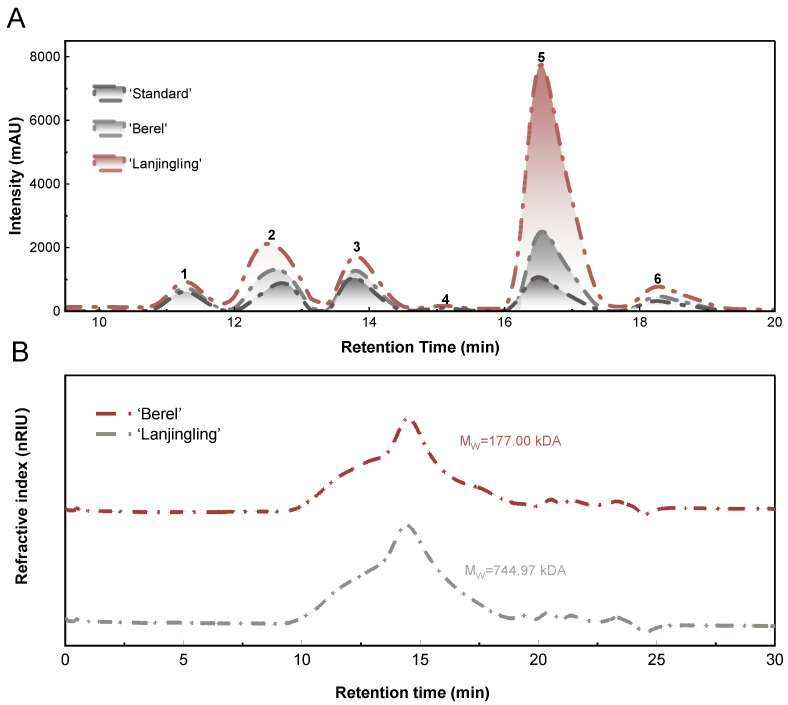
(**A**) Chromatographic profiles of standard monosaccharides and blue honeysuckle polysaccharides: (1) glucose, (2) galactose, (3) rhamnose, (4) mannose, (5) arabinose, and (6) galacturonic acid; (**B**) molecular weight and distribution of ‘Berel’ and ‘Lanjingling’ polysaccharides.

The monosaccharide composition of the ‘Berel’ and ‘Lanjingling’ polysaccharides was determined by HPLC, as shown in Figure 3A and Table 1. The ‘Berel’ polysaccharides consisted of arabinose, galacturonic acid, rhamnose, galactose, and glucose in molar ratios of 12.2:8.4:3.9:2.7:1, respectively. In contrast, the corresponding molar ratios in the ‘Lanjingling’ polysaccharides were 27.5 (arabinose):3.3 (galactose):2.1 (galacturonic acid):1.4 (rhamnose):1 (glucose). Thus, although both cultivars shared the same monosaccharide profile, their molar ratios differed markedly.

### 2.4. Molecular Weight Analysis of the Blue Honeysuckle Polysaccharides

As shown in Figure 3B and Table 1, both ‘Berel’ and ‘Lanjingling’ polysaccharides displayed narrow and symmetrical chromatographic peaks, indicating high purity and good homogeneity. The polydispersity index (Pd), serving as a measure of molecular heterogeneity [6], further demonstrated notable differences between the two cultivars. As illustrated in Figure 1A, the Pd of the ‘Lanjingling’ polysaccharides was 5.1-fold higher than that of ‘Berel’, suggesting that the ‘Berel’ polysaccharides possessed greater molecular uniformity.

### 2.5. In Vitro Antioxidant Activities of the Blue Honeysuckle Polysaccharides

Prior studies have validated that polysaccharides from blue honeysuckle berries have been shown to exhibit considerable in vitro antioxidant capacity [4]. Because our assays used Trolox as the reference standard, whereas other reports used ascorbic acid, IC_50_ values were converted into ascorbic acid equivalents (AAEs) for comparison of ABTS^+^ and DPPH scavenging activities. And the IC_50_ values of ascorbic acid correspond to 1.85-fold and 1.65-fold those of Trolox in the ABTS^+^ and DPPH assays, respectively [13]. As presented in Figure 1 and Table 1, the ABTS^+^ and DPPH scavenging activities of the ‘Berel’ polysaccharides (7.36 ± 0.35 mg AAE/g DW and 38.56 ± 0.25 mg AAE/g DW) were approximately 1.32-fold and 1.44-fold higher, respectively, than those of ‘Lanjingling’ (5.59 ± 0.63 mg AAE/g DW and 26.70 ± 1.06 mg AAE/g DW). These results indicate that the antioxidant capacity of blue honeysuckle polysaccharides varies significantly across cultivars.

### 2.6. Starch Digestive Enzyme Inhibition Activities

**Figure 4:**
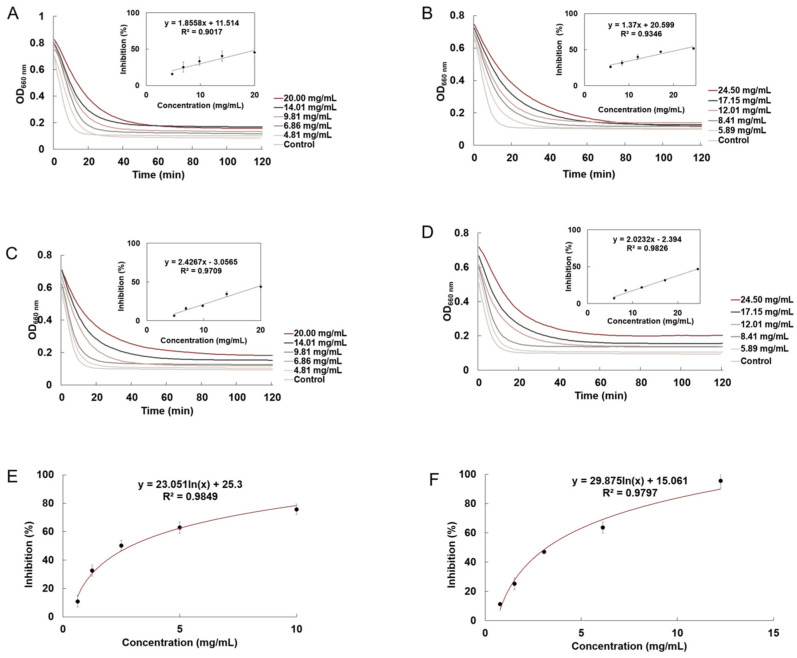
Kinetic curves of inhibition activities of polysaccharides from two cultivars of blue honeysuckle. (**A**) The α-amylase inhibition activity of ‘Berel’ berries; (**B**) the α-glucosidase inhibition activity of ‘Berel’ berries; (**C**) the α-amylase inhibition activity of ‘Lanjingling’ berries; (**D**) the α-glucosidase inhibition activity of ‘Lanjingling’ berries; (**E**) the lipase inhibition activity of ‘Berel’ berries; and (**F**) the lipase inhibition activity of ‘Lanjingling’ berries.

The differences in α-amylase and α-glucosidase inhibition activities between the ‘Berel’ and ‘Lanjingling’ polysaccharides are presented in Figure 1 and Figure 4 and Table 1. The IC_50_ values of the two polysaccharides against starch-digesting enzymes were subsequently expressed as acarbose equivalents (AE). The IC_50_ value of α-amylase inhibition by the ‘Berel’ polysaccharides (2.80 ± 0.32 mg AE/g DW) was slightly higher than that of ‘Lanjingling’ (2.79 ± 0.05 mg AE/g DW). In contrast, the IC_50_ value for α-glucosidase inhibition by the ‘Berel’ polysaccharides (1.55 ± 0.07 mg AE/g DW) was significantly higher than that of ‘Lanjingling’ (1.32 ± 0.06 mg AE/g DW).

### 2.7. Lipase Inhibition Activities

The lipase inhibition activities of the ‘Berel’ and ‘Lanjingling’ polysaccharides are presented in Figure 4E,F and Table 1. For consistent comparison, the inhibition activities were further expressed as orlistat equivalents (OE). The ‘Berel’ polysaccharides exhibited significantly stronger lipase inhibition (17.52 ± 0.61 mg OE/g DW) than the ‘Lanjingling’ polysaccharides (15.28 ± 0.54 mg OE/g DW).

### 2.8. Correlation Analysis of Physicochemical Properties and Bioactivity

**Figure 5:**
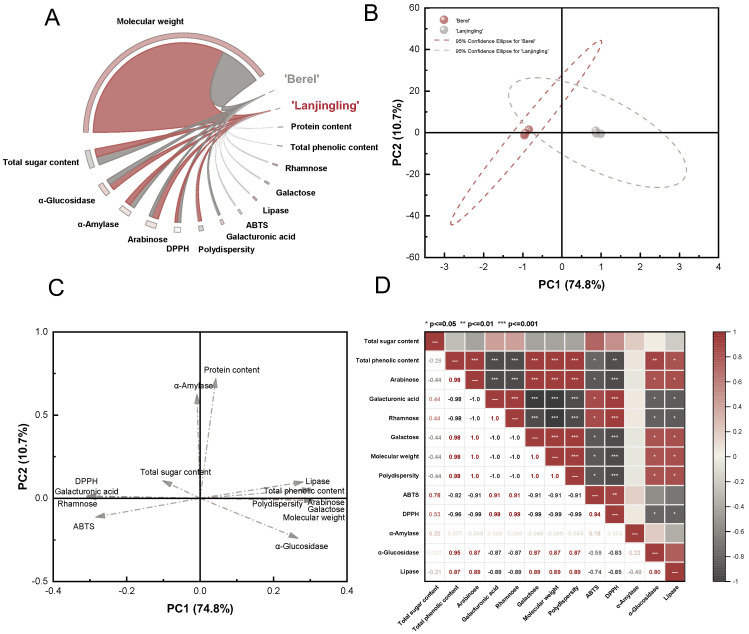
(**A**) Circos plot showing associations between fourteen characteristics and ‘Berel’ and ‘Laningling’ polysaccharides; (**B**) principal component analysis (PCA) plot showing associations of ‘Berel’ and ‘Lanjingling’ polysaccharides; (**C**) PCA plot showing associations of characteristics among blue honeysuckle polysaccharides; and (**D**) correlation analysis of bioactivities and physicochemical properties among blue honeysuckle polysaccharides based on Pearson correlation (* *p* < 0.05, ** *p* < 0.01, and *** *p* < 0.001).

A Circos plot was generated to visualize the associations among fourteen characteristics of the ‘Berel’ and ‘Lanjingling’ polysaccharides. As shown in Figure 5A, MW emerged as the most critical factor distinguishing the two cultivars, further supporting the conclusion that blue honeysuckle cultivar type can substantially influence polysaccharide MW.

To uncover internal relationships among physicochemical and biological characteristics, correlation analysis was performed across chemical properties, molecular weight and distribution, monosaccharide composition, antioxidant activity, and digestive enzyme inhibition. Among these variables, MW and polydispersity (Pd) were key indicators. As shown in Figure 5D, MW and Pd were positively correlated with arabinose and galactose (r = 1, *p* < 0.001). When combining molecular weight parameters with monosaccharide composition to explore their influence on antioxidant capacity, arabinose, galactose, MW, and Pd showed strong negative correlations with ABTS^+^ (r = −0.91, *p* < 0.05) and DPPH (r = −0.99, *p* < 0.001). Conversely, rhamnose and galacturonic acid exhibited significant positive correlations with ABTS^+^ and DPPH. Regarding digestive enzyme inhibition, arabinose, galactose, MW, and Pd were positively correlated with α-glucosidase (r = 0.87, *p* < 0.05) and lipase inhibition (r = 0.89, *p* < 0.05), whereas rhamnose and galacturonic acid were negatively correlated (r = −0.87 and −0.89, respectively; *p* < 0.05). Total phenolic content, another highly relevant parameter, exhibited strong positive correlations with arabinose, galactose, MW, Pd (r = 0.98, *p* < 0.001), α-glucosidase (r = 0.95, *p* < 0.01), and lipase inhibition (r = 0.87, *p* < 0.05). In contrast, it showed significant negative correlations with rhamnose and galacturonic acid (r = −0.98, *p* < 0.001), ABTS^+^ (r = −0.82, *p* < 0.05), and DPPH (r = −0.96, *p* < 0.01).

### 2.9. Principal Component Analysis (PCA)

PCA was performed to elucidate the association between the two blue honeysuckle polysaccharides and fourteen characteristics encompassing physicochemical properties and bioactivities. Scores from the first two principal components (PC1 and PC2) (Figure 5B) were plotted to illustrate sample clustering and distribution. ‘Lanjingling’ polysaccharides showed a strong influence on PC1, whereas ‘Berel’ polysaccharides contributed more prominently to PC2. As shown in Figure 5C, total phenolic content, arabinose, galactose, MW, Pd, α-glucosidase, and lipase activities were positively associated with PC1.

## 3. Discussion

### 3.1. Chemical Composition of Blue Honeysuckle Polysaccharides

Previous studies on blue honeysuckle polysaccharides often used unspecified cultivars, underscoring the need for comparative analyses among defined varieties. For instance, the total sugar content reported for purified polysaccharides extracted from blue honeysuckle berries collected in Mudanjiang, China (129°63′32″ E, 44°55′17″ N) was 43.60 ± 0.83%, which is 1.26-fold higher than that of our ‘Lanjingling’ polysaccharides. This discrepancy may be attributed to the use of ultrasonic cell disruption in that study [8]. Ultrasonic treatment induces cavitation, generating high shear forces and localized heating that promote polysaccharide depolymerization, thereby altering their bioactivity [14]. Because our objective was to compare polysaccharides across cultivars, we deliberately avoided ultrasonic-assisted extraction. Overall, these results indicate that while cultivar differences influence the total sugar content of blue honeysuckle polysaccharides, the extraction and purification methods exert a more substantial impact [15].

### 3.2. FT-IR Spectrum of the Blue Honeysuckle Polysaccharides

The phenomenon demonstrates that the ‘Berel’ and ‘Lanjingling’ polysaccharides are acidic heteropolysaccharides with similar fundamental structural properties, consistent with reports on the same cultivars grown at Northeast Agricultural University (126°32′11″ E, 45°24′15″ N), China [4].

### 3.3. Monosaccharide Composition Analysis

To further explore the influence of cultivar variation on monosaccharide composition, we compared our findings with previous studies. Similar predominant monosaccharides—galactose, arabinose, and galacturonic acid—were reported in polysaccharides extracted from blue honeysuckle berries cultivated in Mudanjiang (129°86′46″ E, 44°56′49″ N) and Shangzhi (128°76′81″ E, 45°16′13″ N), China [5,8]. However, while arabinose was the dominant monosaccharide in both ‘Berel’ and ‘Lanjingling’ in our study, galactose was the major component in samples from Mudanjiang and Shangzhi [5,7,8]. Moreover, even polysaccharides isolated from the same ‘Berel’ cultivar have been shown to exhibit different molar ratios [4,6], indicating notable compositional variability.

Overall, these findings indicate that the monosaccharide proportions of blue honeysuckle polysaccharides are not determined solely by cultivar type but are also affected by environmental and agronomic factors such as climate, soil nutrient availability, and harvest year.

### 3.4. Molecular Weight Analysis of the Blue Honeysuckle Polysaccharides

Weight-average molecular weight (MW) could show different degrees of aggregation of polymers in blue honeysuckle polysaccharides [16,17]. The MW of ‘Berel’ polysaccharides (177.00 kDa) was 4.2 times less than ‘Lanjingling’ (744.97 kDa). Additionally, previously reported MW values for polysaccharides extracted from the same two cultivars cultivated at Northeast Agricultural University (105.60 kDa and 85.30 kDa for ‘Berel’ and ‘Lanjingling’, respectively) were markedly lower than those obtained in our study, 1.68-fold and 2.08-fold lower for ‘Berel’, and 7.05-fold and 8.73-fold lower for ‘Lanjingling’ [4,6]. The noted differences are likely a consequence of sonication-induced depolymerization of the polysaccharide chains, as well as differences in test materials (e.g., pomace vs. fresh berries). Overall, the results indicate that blue honeysuckle cultivar type influences polysaccharide MW, while extraction methodology and sample source further contribute to variability.

### 3.5. In Vitro Antioxidant Activities of the Blue Honeysuckle Polysaccharides

When compared with polysaccharides extracted from ‘Berel’ cultivated at Northeast Agricultural University, the ABTS^+^ and DPPH scavenging activities of our ‘Berel’ samples were 19.37-fold and 350-fold higher (0.38 mg AAE/g DW and 0.11 mg AAE/g DW), likely due to differences in harvest year (2023 vs. 2020) [6]. Conversely, polysaccharides from ‘Lanjingling’ pomace reported in the literature exhibited markedly higher antioxidant activity (250.00 mg AAE/g DW and 653.85 mg AAE/g DW), 44.72-fold and 40.41-fold higher than our ‘Lanjingling’ samples, and 33.97-fold and 27.98-fold higher than our ‘Berel’ samples [4]. This substantial enhancement is likely attributable to polysaccharide degradation in pomace following polyphenol extraction, which can improve antioxidant capacity. Overall, the results reveal that cultivar type is crucial to determine antioxidant activity, and the ‘Berel’ polysaccharides show stronger ABTS^+^ and DPPH scavenging activity. Thus, ‘Berel’ may be more suitable for applications targeting antioxidant functionality.

### 3.6. Starch Digestive Enzyme Inhibition Activities

As ‘Berel’ is the predominant and well-studied blue honeysuckle cultivar in China, the α-amylase and α-glucosidase inhibition activities of our ‘Berel’ polysaccharides were 12.73 and 15.50 times stronger than those reported for ‘Berel’ polysaccharides cultivated at Northeast Agricultural University in China (0.22 mg AE/g DW and 0.10 mg AE/g DW) [6]. However, the α-amylase and α-glucosidase inhibition activities of polysaccharides from ‘Lanjingling’ cultivated at the same institution (300 mg AE/g DW and 277.78 mg AE/g DW) were approximately 108-fold and 210-fold stronger than our ‘Lanjingling’ polysaccharides, and about 107-fold and 179-fold stronger than our ‘Berel’ polysaccharides [4]. These pronounced discrepancies likely stem from differences in test materials: the other group extracted ‘Berel’ polysaccharides from untreated berries, whereas ‘Lanjingling’ polysaccharides were isolated from pomace following polyphenol extraction. Overall, these results reveal that ‘Berel’ polysaccharides may be more suitable for hypoglycemic applications due to their ability to inhibit starch-digesting enzymes.

### 3.7. Lipase Inhibition Activities

Because only limited studies have evaluated the lipase inhibition activities of blue honeysuckle polysaccharides with orlistat as the positive control, we compared the inhibition activities of ‘Berel’ and ‘Lanjingling’ with those of aqueous extracts from other small berries to assess their potential in reducing fat absorption and accumulation [18]. The lipase inhibition activity of ‘Berel’ polysaccharides was 92 and 515 times higher than that of sea buckthorn (0.19 mg OE/g DW) and rowanberry (0.03 mg OE/g DW) aqueous extracts, respectively [19]. Additionally, the IC_50_ values of the ‘Berel’ polysaccharides were more than 2190 times stronger than those reported for black chokeberry (0.008 mg OE/g DW) and elderberry (0.002 mg OE/g DW) aqueous extracts [19]. Overall, these findings suggest that polysaccharides from the ‘Berel’ berry show strong potential for application in anti-obesity food products aimed at reducing fat absorption and lipid accumulation.

### 3.8. Correlation Analysis of Physicochemical Properties and Bioactivity

Monosaccharide composition and molar ratios strongly influence molecular weight and distribution. This finding was corroborated [20] and emphasized that the structural units and branching patterns of polysaccharides are determined by their monosaccharide composition. Arabinose, galactose, MW, and Pd showed strong negative correlations with ABTS^+^ and DPPH, while rhamnose and galacturonic acid exhibited significant positive correlations with ABTS^+^ and DPPH. These observations align with Pei et al. [6], who reported that high uronic acid content enhances radical scavenging activity. Xu et al. [21] also noted that polysaccharides of reduced molecular weight exhibited higher DPPH scavenging activity, as observed in degraded blackcurrant polysaccharides (88.27 ± 2.73% and 95.16 ± 0.14% at 1.2 mg/mL with MWs of 1.30 × 10^4^ kDa and 9.62 × 10^3^ kDa, respectively). Conversely, Ahmadi et al. [15] revealed that raspberry polysaccharides with low MW (637.8 kDa) showed the weakest DPPH scavenging activity. Together, these results suggest that strong antioxidant activity may stem from a high galacturonic acid proportion and an appropriate MW range rather than simply high or low MW alone. Consistent with our findings, Pei et al. [4] reported a positive correlation between α-glucosidase inhibition and MW, while Fu et al. [8] observed a negative correlation between α-glucosidase inhibition and uronic acid content. The phenomenon suggests that polyphenols may enhance MW through increased hydrogen bonding and covalent interactions, and the polysaccharide-polyphenol complexes may provide additional active sites for interacting with α-glucosidase and lipase, thereby improving inhibition activities [15]. In summary, the bioactivities of blue honeysuckle polysaccharides are attributed to their structural properties, including MW and Pd, as well as monosaccharide composition and molar ratios.

### 3.9. Principal Component Analysis (PCA)

The partial overlap of the two cultivar groups indicates a limited degree of similarity, supporting the conclusion from chemical composition analysis that cultivar was not a major determinant of total polysaccharide content. Given the strong loading of ‘Lanjingling’ polysaccharides on PC1, these properties were most characteristic of the ‘Lanjingling’ cultivar. In contrast, protein content and α-amylase activity contributed positively to PC2, indicating that ‘Berel’ polysaccharides exhibited higher protein content and a higher IC_50_ value for α-amylase inhibition.

In summary, while our study provides essential information on monosaccharide composition, molecular weight, and functional group characteristics, we acknowledge that detailed structural features such as glycosidic linkage types and branching patterns remain to be resolved. Future studies employing NMR spectroscopy and GC-MS linkage analysis are warranted to establish a more comprehensive structure–activity relationship for blue honeysuckle polysaccharides.

## 4. Materials and Methods

### 4.1. Materials

Mature fruits in blue honeysuckle cultivars ‘Berel’ and ‘Lanjingling’ were collected in June 2022 from the Trial Farm of Northeast Agricultural University in Harbin, Heilongjiang Province, China (126°32′11″ E, 45°24′15″ N). Glucose, rhamnose, galactose, arabinose, mannose, and galacturonic acid were purchased from Aladdin Co. (Shanghai, China). Ethanol, ether, and acetone were obtained from Kemiou Chemical Reagent Co. (Tianjin, China). Dialysis bags and bovine serum albumin were supplied by Solarbio Science & Technology Co. (Beijing, China). Folin–Ciocalteu phenol reagent was sourced from Bomei Biotechnology Co. (Hefei, China).

### 4.2. Isolation and Purification of Polysaccharides from Blue Honeysuckle

The isolation process of the ‘Berel’ and ‘Lanjingling’ polysaccharides was conducted based on the method of Wang et al. [22], with slight modifications. Fresh fruits of blue honeysuckle were lyophilized for 48 h using a lyophilizer (VaCo-2, ZIRBUS, Bad Grund, Germany) to obtain freeze-dried powder. To remove impurities, including pigments and phenolic compounds, the powder was extracted with 95% ethanol five times. The resulting residue was passed through a 30 mm mesh and subsequently dried in a vacuum oven (DHG-9076A, JINGHONG, Shanghai, China). After 2 h of extraction in water (1:15 *w*/*v*, 90 °C), the dried material was clarified by centrifugation for 10 min at 6000× *g* (Sorvall ST4R Plus, Thermo Fisher Scientific, Waltham, MA, USA). The precipitate was subjected to two additional cycles of hot-water extraction and centrifugation to maximize polysaccharide recovery. The combined supernatants were condensed to one-tenth of the original volume by a RE-52 rotary evaporator (YARONG, Shanghai, China). Subsequently, 95% ethanol was slowly added at a 4:1 (*v*/*v*) ratio with continuous stirring. After incubation at room temperature for 12 h, visible flocculent precipitates formed and were centrifuged to harvest. The precipitate was washed sequentially with ethanol and ether to remove residual water, and with acetone to eliminate remaining organic solvents. The resulting material constituted the crude polysaccharide extract.

Protein removal was performed using the Sevag method after redissolving the crude extract in distilled water [23]. The mixture was subjected to vigorous shaking for 30 min, followed by centrifugation to remove the denatured protein layer. This process was repeated three times to ensure complete protein removal. The solution was then condensed to one-fifth of its initial volume and dialyzed for 48 h using a 7 kDa molecular weight cutoff membrane. The purified polysaccharide solution was finally lyophilized and ground into a homogeneous powder for subsequent analyses.

### 4.3. Physicochemical Properties of Polysaccharides

#### 4.3.1. Chemical Components Analysis

Protein content was determined at 590 nm using bovine serum albumin as the standard [24]. The total sugar content of the purified polysaccharide solution was determined using the phenol-sulfuric acid method at 490 nm against a D-glucose standard [25]. Total phenolic content was quantified at 760 nm, with gallic acid equivalents as the reference [26].

#### 4.3.2. Fourier Transform Infrared Spectroscopy (FT-IR)

The blue honeysuckle polysaccharides were mixed with KBr and pressed into pellets for analysis by an IRTracer-100 FT-IR spectrophotometer (SHIMADZU, Kyoto, Japan). The FT-IR spectrum was acquired over the region of 400–4000 cm^−1^ [22].

#### 4.3.3. Monosaccharide Composition Analysis Using High-Performance Liquid Chromatography with Refractive Index Detection (HPLC-RID)

The polysaccharide powder (10 mg) was hydrolyzed with 2 mL of 4 M TFA, and the reaction was conducted at 110 °C for a duration of 4 h in an oil bath. Residual TFA was eliminated by co-evaporating the hydrolysate on a rotary evaporator with methanol.

Monosaccharide composition was performed with slight adjustments [27]. Prior to a membrane filtration (0.22 μm), the hydrolyzed sample was dissolved in distilled water. Following this procedure, a 2.4 μL injection was loaded onto an Agilent Ca column (7.7 × 300 mm, Agilent Technologies, Santa Clara, CA, USA) and detected using a RID (G7115A, Agilent Technologies, Santa Clara, CA, USA) regulated at 85 °C. Ultrapure water was served as the mobile phase at a flow rate of 0.6 mL/min throughout the chromatographic separation. Monosaccharide composition and molar ratios were determined by comparing retention times with six standards: galacturonic acid, glucose, rhamnose, arabinose, mannose, and galactose.

#### 4.3.4. Molecular Weight Analysis Using High-Performance Gel Permeation Chromatography (HP-GPC)

According to Feng et al. [28], the weight-average molecular weights (MWs) of the ‘Berel’ and ‘Lanjingling’ polysaccharides were measured by HP-GPC. Dextran standards and glucose were used for calibration. At a concentration of 1.0 mg/mL in 0.1 M NaNO_3_ (with 0.02% NaN_3_), the polysaccharides were subjected to a membrane filtration (0.45 μm). Analysis was conducted on a 1260 HPLC system equipped with a RID (G1362A, Agilent Technologies, Santa Clara, CA, USA) and Waters Ultrahydrogel™ columns (Linear: 7.8 × 300 mm; Guard: 6 × 40 mm) (Waters, Milford, MA, USA) at 35 °C. Chromatographic runs were performed using a mobile phase composed of 0.1 M NaNO_3_ and 0.02% NaN_3_, which was delivered at a constant flow rate of 0.6 mL/min.

### 4.4. Antioxidant Activities

#### 4.4.1. DPPH Radical Scavenging Activity

The DPPH radical scavenging activity of the blue honeysuckle polysaccharide was quantified following the method [29]. First, 5 µL of sample solution was added to 195 µL of 60 µM DPPH working solution in a microplate well. Subsequently, the plate was covered and incubated for 2 h under light-protected conditions. With a microplate reader (Epoch 2, BioTek, Winooski, VT, USA), the absorbance at 517 nm was measured. A Trolox standard curve was prepared, and antioxidant activity was expressed as milligrams of Trolox equivalents per gram of dry weight (mg TE/g DW).

#### 4.4.2. ABTS^+^ Radical Scavenging Activity

The method established by Re et al. [30] was employed to evaluate the ABTS^+^ scavenging activity of the blue honeysuckle polysaccharides. A 10 µL aliquot of polysaccharide solution or Trolox standard was treated with 190 µL of ABTS^+^ solution and incubated for 10 min under light-protected conditions. The antioxidant capacity was determined based on the absorbance reading at 734 nm, and quantified in mg TE/g DW.

### 4.5. Starch Digestive Enzyme Inhibition Activities

With reference to Liu et al. [31], the α-amylase and α-glucosidase inhibition activities of blue honeysuckle polysaccharides were evaluated. A translucent maize starch solution was obtained by adopting phosphate buffer (pH 6.9) as the solvent and heating with stirring. The reaction was initiated by adding 60 µL of the translucent starch solution to a microplate with 96 wells. Subsequently, 20 µL of polysaccharide samples at various concentrations were mixed with 20 µL of α-amylase or α-glucosidase and incubated at 37 °C for 15 min. Following a 2 h reaction period at 37 °C, the absorbance of the mixture was measured at 660 nm. Water and acarbose were served as the blank and positive control, respectively. The inhibition activities of the polysaccharides against α-amylase and α-glucosidase, expressed as IC_50_ values, were calculated using Equations (1) and (2).

(1) $$\mathrm{AUC}=0.5+\frac{f_{2}+f_{n-1}}{f_{2}}$$

(2) $$\mathrm{Inhibition}\left(\%\right)={\mathrm{AUC}}_{1}-\frac{{\mathrm{AUC}}_{0}}{{\mathrm{AUC}}_{1}}$$

where AUC is the total area under the curve; f_1_, f_2_, …, f_n_ represent absorbance values at each measurement time point; AUC_1_ is the area under the inhibition curve of the sample; and AUC_0_ is the area under the curve of the blank control.

### 4.6. Lipase Inhibition Activities

The method of Gilham and Lehner [32] was applied to assess the lipase inhibition activity of the blue honeysuckle polysaccharides, with minor modifications. The buffer solution was formulated by mixing Tris-EDTA (50 mM) with 0.35% (*w*/*v*) sodium deoxycholate, adjusting the pH to 8.3, and storing the solution at 4 °C. Subsequently, 15 mg of p-nitrophenyl phosphate disodium (pNPP) was solubilized in isopropanol to obtain a pNPP solution, which was also stored at 4 °C. Lipase (10 mM) was mixed with the buffer solution, vortexed for 2 min, and centrifuged. The resulting supernatant was collected and stored frozen. And then, 0.1 g of the sample was solubilized in 1 mL of dichloromethane and extracted for 12 h on a shaker at 180 rpm and 25 °C. The supernatant (20 µL) was mixed with 300 µL of buffer in a centrifuge tube and sonicated for 30 min.

Prior to the enzymatic assay, 150 µL of the mixture was transferred to a 96-well microplate and maintained at 37 °C for 15 min. Thereafter, 10 µL of lipase solution was added to commence the reaction, and the plate was incubated for a further 10 min. Lastly, 10 µL of pNPP solution was added, and the reaction was monitored at 410 nm by recording the absorbance at 1 min intervals for 1 h. The lipase inhibition activity of the blue honeysuckle polysaccharides was calculated according to Equation (3).

(3) $$\mathrm{Inhibition}\left(\%\right)=\left(1-\frac{A_{1}}{A_{0}}\right)\times 100$$

where A_1_ is the absorbance of the tested blue honeysuckle polysaccharides; A_0_ is the absorbance of the blank control.

### 4.7. Statistical Analysis

Principal component analysis and Pearson correlation analysis were conducted using Origin 2022 software (version 9.9.5.171, OriginLab, Northampton, MA, USA), with statistical significance defined at three thresholds: * *p* < 0.05, ** *p* < 0.01, and *** *p* < 0.001. Values were reported as mean ± standard deviation from three technical replicates by Excel and significance through SPSS Statistics 19.0 software.

## 5. Conclusions

In this study, polysaccharides from the blue honeysuckle cultivars ‘Berel’ and ‘Lanjingling’ were extracted and systematically compared. Correlation analysis demonstrated that the composition and proportions of monosaccharides, together with molecular weight and its distribution, jointly influenced these polysaccharide bioactivities. These findings provide a new perspective on how cultivar differences shape the structure–activity relationships of polysaccharides from the blue honeysuckle berries. Overall, ‘Berel’ polysaccharides exhibited higher total sugar content, lower molecular weight and polydispersity, and stronger antioxidant capacity, as well as greater inhibitory activity against α-glucosidase and lipase, contrasted with those from ‘Lanjingling’. Thus, ‘Berel’ polysaccharides show strong potential for application in medicinal and functional food products aimed at mitigating postprandial hyperglycemia and reducing lipid accumulation. Nevertheless, further research is warranted to identify additional polysaccharide sources from other blue honeysuckle cultivars and to examine the influence of other factors, such as berry developmental stage, on polysaccharide characteristics and bioactivity.

## Figures and Tables

**Table 1:** Physicochemical properties and bioactivities of blue honeysuckle polysaccharides.

Samples	‘Berel’	‘Lanjingling’
Total sugar content (%)	34.59 ± 1.37 ^a^	32.56 ± 3.28 ^a^
Total phenolic content (%)	0.72 ± 0.04 ^a^	1.03 ± 0.03 ^b^
Protein content (%)	0.81 ± 0.08 ^a^	0.82 ± 0.05 ^a^
Arabinose (molar ratio)	12.2	27.5
Galacturonic acid (molar ratio)	8.4	2.1
Rhamnose (molar ratio)	3.9	1.4
Galactose (molar ratio)	2.7	3.3
Glucose (molar ratio)	1	1
Molecular weight (MW, kDa)	177.00	744.97
Polydispersity (Pd)	2.75	13.98
ABTS^+^ (mg TE/g DW)	3.98 ± 0.19 ^a^	3.01 ± 0.34 ^b^
DPPH (mg TE/g DW)	14.16 ± 0.09 ^a^	9.80 ± 0.39 ^b^
α-Amylase IC_50_ (mg/mL)	21.66 ± 2.80 ^a^	21.50 ± 0.50 ^a^
α-Glucosidase IC_50_ (mg/mL)	21.95 ± 1.27 ^a^	25.87 ± 1.39 ^b^
Lipase IC_50_ (mg/mL)	2.80 ± 0.12 ^a^	3.21 ± 0.14 ^b^

The data is presented as mean ± standard deviation, and different lowercase letters (a and b) indicate significant differences (*p* < 0.05).

## Data Availability

The original data presented in this study are included in the article Further inquiries can be directed to the corresponding author.
